# Non-linear plasma protein binding of cannabidiol

**DOI:** 10.1186/s42238-024-00238-8

**Published:** 2024-06-20

**Authors:** Mariana Babayeva, Iva Srdanovic

**Affiliations:** Department of Biomedical and Pharmaceutical Sciences, Touro College of Pharmacy, 3 Times Square, New York, NY 10036 USA

**Keywords:** Plasma protein binding, Cannabidiol, CBD, Tizoxanide, Ultrafiltration, Non-linear pharmacokinetics

## Abstract

**Background:**

Cannabidiol is highly bound to plasma proteins. Changes in its protein binding can lead to altered unbound plasma concentrations and result in alteration of pharmacological activity of cannabidiol-containing medications. This research has assessed non-linearity of cannabidiol plasma protein binding and the potential effect of tizoxanide on the binding.

**Method:**

Cannabidiol protein binding was evaluated by ultrafiltration technique. Human plasma was spiked with cannabidiol stock solution to produce samples of various concentrations. For interaction study potential interactant tizoxanide was added in each sample. All samples were processed through Amicon Micropartition system and analyzed by HPLC.

**Results:**

The study has detected cannabidiol binding to borosilicate glass (9%) and polyethylene plastics (15%). In the interaction study the mean protein unbound fraction of cannabidiol was 0.05 (5%), indicating no binding interaction between cannabidiol and tizoxanide since cannabidiol unbound fraction without tizoxanide was also 5%. The cannabidiol fraction unbound was more than 2-fold greater at high concentrations compared to low concentrations.

**Conclusion:**

a). At high concentrations cannabidiol plasma protein binding is non-linear. The non-linearity can affect elimination and medicinal effect of cannabidiol drugs. b). Borosilicate and polyethylene containers should be avoided in formulation, packing and administration of cannabidiol-containing medicines to guarantee correct doses. c). Cannabidiol medications can be co-administered with tizoxanide without caution.

## Background

Cannabis plant holds nearly 100 cannabinoids. Two of them, cannabidiol (CBD) and Δ^9^-tetrahydrocannabinol (THC) are well studied. THC is the primary psychotropic ingredient of cannabis. In contrast, CBD does not have psychoactive properties. Another nonpsychotropic cannabinoid cannabidivarin (CBDV) is similar to CBD structurally and functionally. CBD and CBDV demonstrate anti-inflammatory, antioxidant, anticonvulsant, and neuroprotective properties Alexander et al. (2018); Babayeva and Loewy (2016); Babayeva et al. (2016) and are favorable medicinal candidates for treatment of epilepsy, Alzheimer’s, Parkinson’s diseases, and other neurological disorders Babayeva et al. (2022); Badowski (2017); Celestin and Musteata (2021). Epidiolex® (CBD) was FDA-approved for epilepsy-associated Dravet and Lennox-Gastaut syndromes Drugbank Cannabidivarin (2023). Sativex® (nabiximols) containing both CBD and THC (1:1) is available in more than 25 nations EMC (2023). In the USA, nabiximols is a trial medicine for polyneuropathy, HIV-related neuropathy, and cancer pain Evoli (2016). THC-containing medications dronabinol and nabilone were approved for the treatment of chemotherapy-induced nausea, vomiting and anorexia FDA news release (2023). CBDV was given orphan designation for treatment of Rett Syndrome and Fragile X Syndrome Celestin and Musteata (2021).

The approval of cannabis-containing drugs has led to a need for inclusive knowledge of cannabinoids pharmacokinetics (PK). PK of highly protein bound drugs, such as CBD, can be affected by alteration in their plasma protein binding. For most drugs, protein binding is linear, and the unbound (free) fraction is relatively constant. However, saturation of binding site or displacement of a drug from its protein site may lead to an increase in unbound fraction with consequent change in pharmacological effect.

Cannabidiol was of particular interest for this investigation since CBD is highly bound to plasma proteins (95%) Furgiuele et al. (2021). Only 5% of absorbed CBD dose can access its active site and produce pharmacologic effect. Tizoxanide is also highly bound to plasma proteins (> 99%) and may have the potential to inhibit CBD plasma protein binding Liu et al. (2022). Tizoxanide is an active metabolite of a broad-spectrum antiparasitic and antiviral prodrug nitazoxanide (Alinia®), which transforms to tizoxanide within a few minutes after administration Calder?n et al. (2020); Morano et al. (2016). In earlier studies tizoxanide significantly reduced plasma protein binding of phenytoin and warfarin Liu et al. (2022); Mullokandov et al. (2014). It was concluded caution should be used to coadminister nitazoxanide with other highly protein-bound medications. Both drugs, CBD and tizoxanide are expected to be co-administered clinically.

Protein binding drug: drug interaction or non-linear binding can increase free plasma concentrations and affect medicinal outcomes of CBD-containing medications. Changes in CBD binding may also affect cannabidiol elimination. In addition, CBD is susceptible to metabolic drug interactions. Cannabidiol inhibits CYP2D6, 2C9, 2C19, 2B6, 2J2, 3A4/5/7 and non-CYP enzymes, UGT1A9 and UGT2B7 Jasenosky et al. (2019). Medicines that are substrates for these enzymes may be at risk of altered elimination by concurrent CBD intake Jasenosky et al. (2019); Lara et al. (2022). Elevated levels of unbound CBD may result in more excessive inhibition of the enzymes and too high plasma concentrations of the affected medications, which may lead to exaggerated pharmacological effect and even toxicity of the drugs.

Estimation of alteration in CBD plasma protein binding is important in drug development and clinical practice. No data were reported on linearity of CBD protein binding or CBD binding interaction with other medications. The goals of this study were: (a) to assess linearity of CBD plasma protein binding and (b) to screen for the potential impact of tizoxanide on CBD plasma protein binding.

## Materials and methods

CBD, DMSO and phosphoric acid were obtained from Sigma Aldrich (St. Louis, MO). Tizoxanide was obtained from TCI Brand (Tokyo, Japan). Methanol was purchased from Fischer Scientific (Pittsburg, PA). HPLC nylon filter (0.45-micron, 47 mm) was purchased from Pall Life Science (Port Washington, NY). Human plasma was purchased from Valley Biomedical (Winchester, VA). Amicon Centrifree YM-30 (molecular weight cut off 30 K) centrifugal filter devices were obtained from Millipore Corporation (Billerica, MA, USA).

The effect of tizoxanide on CBD plasma binding was conducted over a range of CBD concentrations. Human plasma was spiked with CBD stock solution to produce samples of the following concentrations: 100 µg/mL, 150 µg/mL, 200 µg/mL, 250 µg/mL, and 300 µg/mL. A stock solution of tizoxanide was prepared at a concentration of 5 mg/mL. Then CBD-containing aliquots were spiked with 100 µL tizoxanide stock solution each.

Non-linearity study was performed over a range of high CBD concentrations. Human plasma was spiked with CBD stock solution to produce samples of the following concentrations: 300 µg/mL, 350 µg/mL, and 400 µg/mL.

CBD binding was investigated using a centrifugal ultrafiltration method. The investigation was performed at physiologic temperature and pH. The samples were allowed to equilibrate in shaker bath at 37 °C for 30 min. An aliquot of each sample (1 mL) was added to Amicon Centrifree Micropartition System (MW cutoff 30,0000). The system was centrifuged for 30 min at 2000 rpm. The ultrafiltrate was assayed for determination of unbound CBD concentrations. All experiments were performed in triplicate.

CBD unbound concentrations were measured by a novel HPLC method. Separation was accomplished using a Phenomenex C18 column (250 × 4.6 mm, 5 μm) through isocratic elution with a pre-column filter. The column was temperature controlled at 30 °C. The mobile phase was 10% phosphate buffer (0.1% phosphoric acid, pH 2.16) and 90% methanol. Other HPLC variables included a flow rate of 0.9 mL/min, UV wavelength of 220 nm, injection volume of 40 µL. CBD retention time was 5.2 min.

The CBD fraction unbound was calculated as a ratio of unbound concentration to total CBD concentration. Mean estimates of CBD protein binding parameters were compared to corresponding parameters of a previous study when CBD was administered alone and to binding parameters of the lower concentrations. T-tests were used to estimate significant differences.

## Results

Assessment of CBD protein binding was conducted in human plasma. Preliminary studies estimated CBD binding to plastics of ultrafiltration devices and glassware. CBD binding to the polyethylene plastic and to the borosilicate glass was 15% and 9%, respectively. These data were applied in calculation of CBD plasma protein binding.

The CBD protein binding data in the presence of tizoxanide are presented in Table 1.

**Table 1:** Plasma protein binding of CBD in the presence of Tizoxanide

CBD Total concentration	CBD Unbound fraction*
100 µg/mL	0.06 (0.001) 6%
150 µg/mL	0.045 (0.0007) 4.5%
200 µg/mL	0.042 (0.001) 4.2%
250 µg/mL	0.048 (0.002) 4.8%
300 µg/mL	0.105 (0.005) 10.5%

*Data reported as mean (SD)

In the CBD-tixozanide interaction study the mean unbound fraction of CBD was 0.05 (5%) for concentrations range 100–250 µg/mL. No significant difference in the plasma protein binding was found within the concentration group. Similar CBD plasma protein binding (5%) was observed in our earlier study when CBD was administered alone Furgiuele et al. (2021). However, unbound fraction of 300 µg/mL concentration was more than 2-fold greater compared to lower concentrations indicating non-linear plasma protein binding. It was decided to conduct a dose-escalation study to verify non-linear plasma protein binding of CBD.

The CBD protein binding data in the dose-escalation study are presented in Table 2.

**Table 2:** Plasma protein binding of CBD over concentrations from 300 to 400 µg/mL

CBD Total concentration	CBD Unbound fraction*
300 µg/mL	0.107 (0.008) 10.7%
350 µg/mL	0.114 (0.007) 11.4%
400 µg/mL	0.118 (0.009) 11.8%

*Data reported as mean (SD)

In the dose-escalation study the mean CBD unbound fraction was 0.113 (11.3%), which is 2.3-fold higher than mean free fraction at lower concentrations.

## Discussion

This research evaluated the non-linearity of CBD binding and the potential plasma protein binding interaction between CBD and tizoxanide.

Co-administration of tizoxanide did not change plasma protein binding of cannabidiol since analogous CBD binding was observed in our earlier study when CBD was administered alone Furgiuele et al. (2021). Human plasma contains different proteins, among which human serum albumin (HAS) is the main protein interacting with drugs Nasrin et al. (2021). A study has found that CBD binding to albumin was 97.4% Yang et al. (2014). Tizoxanide also has a strong affinity for albumin since its protein binding reached more than 95% in albumin solution, while in the solution of ὰ-1-acid-glycoproteins, the value was only about 49% Pearlson (2020). HSA has two sites where predominantly most drugs bind, either site I in domain II or site II in domain III Nasrin et al. (2021); Pearlson (2020). A complete map of the binding sites of drugs in albumin is difficult to obtain, because of the structural adaptability of this protein in accommodating small ligands Pisanti et al. (2017). Studies have found that tizoxanide mostly binds to albumin site I Mullokandov et al. (2014); Pisanti et al. (2017). Whereas CBD more likely binds to site II as has a more favorable free binding energy for site II over site I with values − 7.5 kcal/mol and − 6.8 kcal/mol, respectively Yang et al. (2014). CBD and tizoxanide, although highly bound to albumin, have an affinity to different sites.

Interestingly, in the interaction study CBD unbound fraction for concentration of 300 µg/mL was more than 2-fold greater than free fractions of the lower concentrations. The finding could be explained by saturation of plasma protein binding at high CBD level. It was decided to conduct a dose-escalation investigation to confirm or to decline non-linear plasma protein binding of CBD. The additional study confirmed that CBD has non-linear plasma protein binding, since the free fractions were 2.3-fold greater at high concentrations compared to lower levels. Such elevated unbound fractions result in much higher free CBD concentrations in the blood and can cause exaggerated pharmacological effect and/or toxicity of CBD-containing medications. This discovery is important as overlooking non-linearity may produce unpredictable results not only for CBD drugs but also for some co-administered medications since CBD is prone to metabolic drug interactions Jasenosky et al. (2019). Increased levels of unbound CBD might result in risky inhibition of metabolism of the affected medications and may lead to exaggerated pharmacological effect and even toxicity of these drugs.

Additionally, now CBD exists in different strengths in the forms of gummies, oils, candies, etc. FDA does not regulate these formulations. The high CBD doses in the preparations can produce high blood concentrations, which may lead to saturation of CBD plasma protein binding and subsequent undesirable effects.

Besides, plasma protein/albumin levels may be reduced by numerous factors, such as stress, surgery, liver, or kidney disfunctions, and pregnancy. Decreased concentrations of albumin may lead to lowered CBD binding. In this case saturation of the protein binding can occur at much lower CBD blood concentrations and may require dose adjustment Zhao et al. (2010).

## Conclusion

The initial discovering demonstrated CBD binding to borosilicate glass and polyethylene plastic. Containers made from these materials should not be used in formulation, packing and administration of CBD-containing drugs to guarantee correct doses of the medications.

CBD as a highly plasma protein-bound drug might be involved in protein binding drug: drug interaction. However, tizoxanide did not alter CBD binding, suggesting that CBD-containing medications can be co-administered with tizoxanide without caution.

Unbound CBD fractions at high concentrations were significantly higher compared to lower concentrations. This discovery suggests non-linear protein binding, which may lead to non-linear pharmacokinetics of CBD. More research is required to estimate the effect of the binding non-linearity on CBD metabolism and excretion.

## Data Availability

The datasets generated and/or analyzed during the current study are available in publicly available repositories.

## Abbreviations

CBD: Cannabidiol
THC: Delta-9-tetrahydrocannabinol
CBDV: Cannabidivarin
HAS: Human albumin solution
